# Evaluating Prognosis and Prognostic Factors in Critically Ill Patients With and Without Cancer: An Observational Comparative Study

**DOI:** 10.7759/cureus.96493

**Published:** 2025-11-10

**Authors:** Mohammed Al Khamis, Maram Aljishi, Albatool Busbaih, Raja Al Dandan, Abdul Salam, Hadi Al Yousef

**Affiliations:** 1 Critical Care Department, King Fahad Specialist Hospital, Dammam, SAU; 2 Internal Medicine Department, King Fahad Specialist Hospital, Dammam, SAU; 3 Oncology Department, King Fahad Specialist Hospital, Dammam, SAU; 4 Epidemiology and Biostatistics Administration, Population Health Management, King Fahad Specialist Hospital, Dammam, SAU

**Keywords:** cancer, critical care, mortality, prognosis, saudi arabia

## Abstract

Objective

There is a lack of published data on the prognosis and factors predicting mortality in the intensive care unit (ICU) for cancer patients in our region. This study aims to examine the outcomes and factors affecting mortality in cancer patients admitted to the ICU in the Eastern Province of Saudi Arabia, in comparison to non-cancer patients.

Materials and methods

This retrospective study analyzed data from patients admitted to a single tertiary center's critical care unit between January 2022 and October 2023. We compared cancer patients (solid and hematological malignancies) to non-cancer patients (admitted under internal medicine), excluding elective cases and those with pre-existing Do Not Resuscitate (DNR) orders.

Results

A study of 313 patients, comprising 165 (53.7%) cancer patients and 148 (47.3%) non-cancer patients, revealed the following findings: Septic shock (74.1%) and respiratory failure (55.3%) were the primary reasons for ICU admission. Among cancer patients, breast cancer (20%) was the most prevalent, followed by colorectal (15.2%) and lung cancers (7.3%). Most oncology patients (78.8%) had metastasis. Median ICU and hospital stays were four and 12 days, respectively, with no significant differences between cancer and non-cancer patients. Cancer patients had significantly higher ICU mortality (58.8% vs. 36.5%) and in-hospital mortality rates (72.7% vs. 43.9%) compared to non-cancer patients. Multivariate analysis identified cancer diagnosis and ischemic heart disease as independent predictors of mortality. Mechanical ventilation was the sole predictor of ICU mortality among cancer patients, while DNR status predicted ICU mortality among non-cancer patients.

Conclusion

Critically ill cancer patients admitted to the ICU face higher mortality rates compared to non-cancer patients despite similar lengths of stay. Invasive mechanical ventilation emerges as a key predictor of mortality among cancer patients. Conversely, cancer diagnosis predicts mortality across both groups. These findings underscore the importance of discussing end-of-life care goals with advanced-stage cancer patients requiring mechanical ventilation.

## Introduction

Cancer poses a pressing global health concern, with a steadily increasing incidence worldwide [1-3]. Rising cases strain healthcare systems, as cancer patients often develop severe complications from the disease or treatment, occupying significant intensive care unit (ICU) capacity and underscoring the importance of focusing on their outcomes [1]. Initially, ICU mortality rates for cancer patients were alarming (1990s reports); however, advancements in medicine, technology, and treatments have improved outcomes [2-6]. Despite this progress, critical care practitioners often prioritize discussing end-of-life care with cancer patients upon ICU admission due to perceived poor prognoses [7]. In contrast, similar discussions are less common for non-cancer patients with comparable prognoses [8]. The presence of a subconscious bias among ICU physicians toward cancer patients was explored in a sub-analysis of the large observational DISPROPRICUS (Disproportionate Care in the ICUs) study. While no such bias was detected, discrepancies remained among ICU physicians in accurately assessing prognosis and making treatment-limitation decisions. Notably, the original study highlighted the importance of fostering a positive ethical climate in ICUs, which has been shown to improve decision-making in such cases [9,10].

The heterogeneity of cancer presentations and patient-specific variables complicates the development of evidence-based ICU triage criteria [11]. A prospective study proposed an ICU admission triage procedure for cancer patients, recommending reassessment of care level by day 5 despite a low overall survival rate (21.8%), organ failure assessment on day 6 better predicted survival than the initial evaluation [12]. Several other studies have identified different predictors of ICU mortality in cancer patients. A Turkish retrospective cohort study found four statistically significant independent predictors: elevated Sequential Organ Failure Assessment (SOFA) scores, septic shock, invasive mechanical ventilation, and acute kidney injury [13]. Another study identified three key predictors of hospital mortality within 180 days: metastatic disease, elevated acute physiology and chronic health evaluation (APACHE II) scores, and sepsis [14]. A retrospective study at Anderson Cancer Center demonstrated the prognostic utility of SOFA scores in predicting acute and long-term survival among metastatic gastrointestinal malignancy patients requiring ICU admission for non-surgical indications [15]. A study conducted across multiple centers in China developed a predictive model that uses eight key indicators to forecast the risk of 90-day mortality in cancer patients who are unexpectedly admitted to the ICU. This model demonstrated high accuracy in predicting outcomes. However, to ensure its reliability and applicability in different healthcare settings and populations worldwide, further global validation is necessary to confirm its effectiveness [16]. A local retrospective study by Rugaan et al. [17] identified SOFA score, invasive ventilation, and vasopressor use as independent mortality predictors in solid tumor patients. Another local study at King Khalid University Hospital in Riyadh analyzed outcomes of 44 patients with hematological malignancies in the ICU and found a significant correlation between remission status, aspartate aminotransferase levels, and patient outcomes. Notably, patients with acute myeloid leukemia (AML) had a very high mortality rate of 90.9% [18]. Two French studies examined outcomes in patients with metastatic solid cancers. The first study found that patients with better performance status, nutritional status, and those receiving targeted treatments were more likely to be discharged home within 90 days. In contrast, factors such as malnutrition, respiratory failure, and higher Simplified Acute Physiology (SAP) scores were associated with increased one-year mortality, which was 71.5% [19]. The second study reported a 30% ICU mortality rate, with surviving patients having a median survival of 173 days. The key predictors of three- and six-month survival included performance status, disease progression, and treatment limitation decisions [20].

The geographical location of cancer patients plays a crucial role in shaping their clinical outcomes, as evidenced by disparities in reported ICU mortality rates between local and international clinical trials [17-23]. This highlights the need to consider regional factors when interpreting and applying trial results, given significant variations in patient disease profiles, family dynamics, and ethical landscapes across centers [9]; despite this, research on ICU mortality and prognosis in cancer patients is scarce, particularly in the Kingdom of Saudi Arabia, with no comparative studies involving non-cancer patients has been published so far. This study aims to bridge this knowledge gap by comparing ICU outcomes and identifying mortality predictors in both cancer and non-cancer patients, ultimately informing evidence-based resource allocation and serving as a foundation for future research.

## Materials and methods

Setting and study design

This single-center, retrospective study was conducted at King Fahad Specialist Hospital, Dammam, Saudi Arabia (IRB approval: ICU0308). The hospital specializes mainly in transplant, oncology, and neuroscience services. Data were gathered from a prospectively collected quality monitoring database and verified through a medical record review. Eligible patients included: age ≥18 years, unplanned ICU admissions under hematology, oncology, or internal medicine services between 2022 and 2023, and first admission only (multiple admissions excluded). We also excluded patients with pre-existing "Do Not Resuscitate" orders prior to ICU admissions, elective postoperative admissions, post-bone marrow transplant patients and COVID-19 diagnoses. Patient outcomes were tracked until hospital discharge or death.

Statistical method

Sample Size

The primary objective of the study was to measure the ICU mortality between two groups (cancer and non-cancer patients). A sample size calculation determined that 152 patients per group would provide sufficient statistical power (90%) to detect a clinically meaningful difference (17.9%) in ICU mortality between cancer (46.2%) and non-cancer (28.3%) patients using a two-tailed z-test/chi-square test (α=0.05) [24].

Statistical Analysis

Descriptive and inferential statistics were employed to characterize the study sample and test hypotheses. The results were reported as mean±standard deviation (SD; for normally distributed data) or median with range (for data not normally distributed), and categorical variables were presented as numbers (percentages).

*Comparative analysis*: Pearson chi-square test compared ICU mortality between cancer and non-cancer patients. Bivariate analysis independent sample t-test, Mann-Whitney U-test, Pearson chi-square test, or Fisher exact test analyzed demographic and clinical differences between solid/hematological malignancy and non-cancer patients. Similar analyses were performed to assess the relationship between demographic data and clinical characteristics with ICU mortality.

The odds ratio and 95% confidence interval were also reported. Multiple binary logistic regression model was used to identify independent factors associated with ICU mortality, adjusting for confounders (e.g., age, sex, primary disease, cancer status). Wald test was used on each factor to determine which factors are significant. Adjusted odds ratio (AOR) and 95% confidence interval for the AOR were reported. Statistical significance was set at P<0.05 (two-tailed). Hosmer-Lemeshow test assessed model goodness-of-fit. All analyses were performed using IBM SPSS Statistics, version 28 (IBM Corp, Armonk, NY).

## Results

Patient characteristics

**Figure 1:**
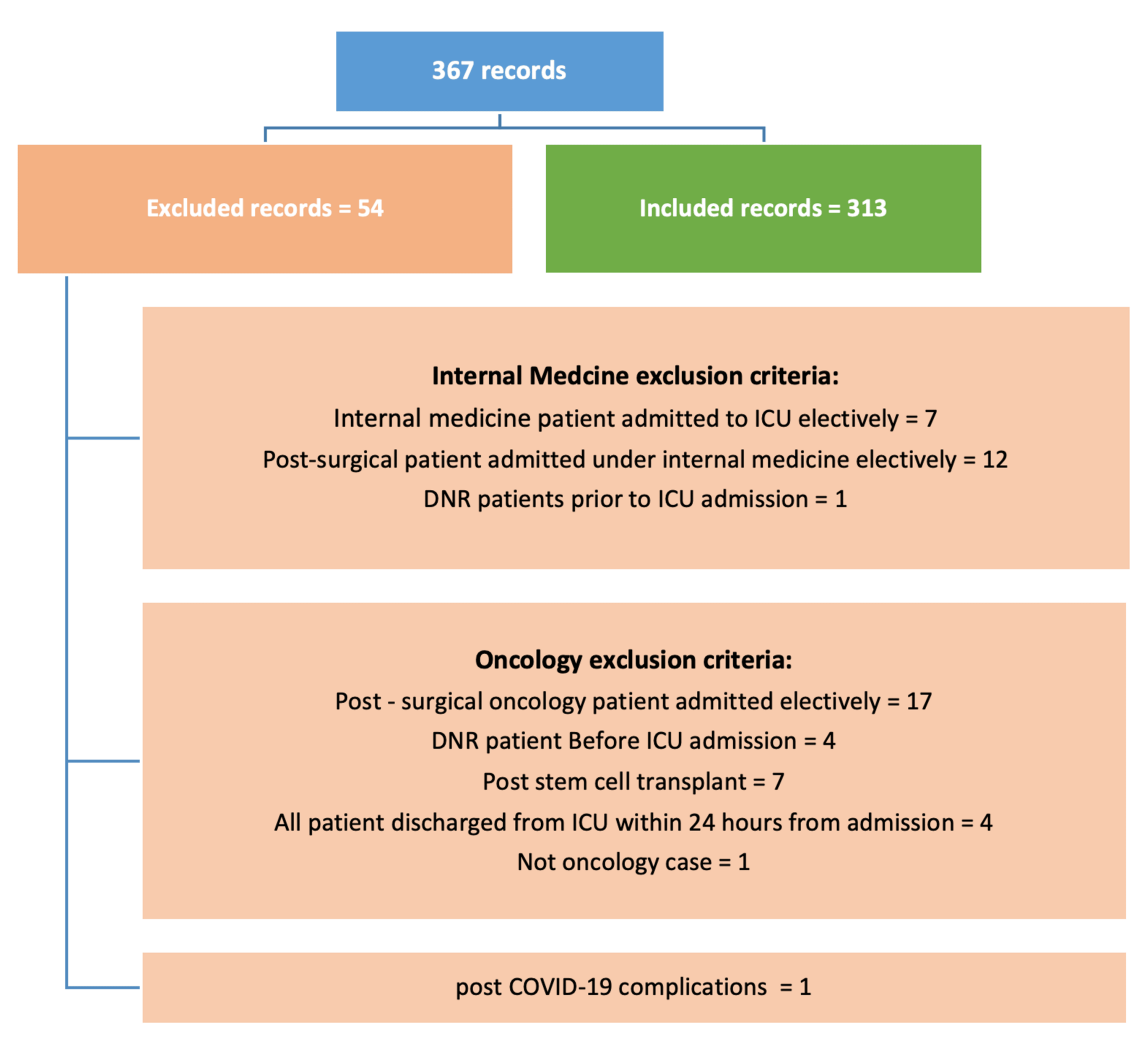
Exclusion Criteria DNR: Do Not Resuscitate.

During the study period, 367 records were reviewed. After applying exclusion criteria (Figure 1), 313 cases remained, comprising 148 (47.3%) IM patients and 165 (52.7%) oncology patients (Table 1). The mean age was 55.93, with oncology patients significantly younger (p=0.014). Men accounted for 160 (51.1%) of the cohort. Most admissions (221, 70.6%) originated from the emergency department, with a higher proportion of medical cases (113, 76.4%) than oncology cases (108, 65.5%) admitted through the ER (p=0.035).

**Table 1:** Relationship between demographic, clinical characteristic and type of patients (IM vs. ON) (n=313). IM: Internal medicine; ON: oncology. Results are expressed as mean±SD, and number (column percentage). ^a^P-value has been calculated using Pearson's chi-square test. ^b^P-value has been calculated using independent sample t-test (Mann-Whitney U test).

Factors	Results	Type of Patients			P-value^a^
		IM (n=148)	ON (n=165)	Test value	
Age in years	55.93±18.33	58.61±20.3	53.52±16.1	-2.475	0.014^b^
Gender				3.572	0.059
Male	160 (51.1%)	84 (56.8%)	76 (46.1%)		
Female	153 (48.9%)	64 (43.2%)	89 (53.9%)		
Systolic blood pressure (SBP)	103.7±27.4	108.26±26.96	99.67±27.23	-2.798	0.005^b^
Diastolic blood pressure (DBP)	59.4±17.2	61.79±17.09	57.21±17.12	-2.359	0.019^b^
Source of admission				4.464	0.035
Non-Emergency Department	92 (29.4%)	35 (23.6%)	57 (34.5%)		
Emergency Department	221 (70.6%)	113 (76.4%)	108 (65.5%)		
Reason for ICU admission					
Respiratory failure	173 (55.3%)	78 (52.7%)	95 (57.6%)	0.749	0.387
Sepsis/septic shock	232 (74.1%)	100 (67.6%)	132 (80.0 %)	6.287	0.012
Neurological disorder	96 (30.7%)	35 (23.6%)	61 (37%)	6.511	0.011
Renal dysfunction	76 (24.3%)	42 (28.4%)	34 (20.6%)	2.563	0.109
Hepatic dysfunction	40 (12.8%)	19 (12.8%)	21 (12.7%)	0.001	0.977

Sepsis and septic shock were the primary reasons for ICU admission (232, 74.1%), followed by respiratory failure (173, 55.3%). Oncology patients had higher rates of sepsis/septic shock (132 (80%) vs 100 (67.6%)) (p=0.012) and neurological disorders (61 (37%) vs 35 (23.6%)) (p=0.011) compared to medical cases. Associated comorbidities and performance status are detailed in Table 2. Notably, ischemic heart disease was more prevalent in non-cancer patients (27 (18.2%) vs 39 (7.3%)) (p=0.003). The cohort's high acuity was reflected in mean APACHE II (25.8±16.4) and Simplified Acute Physiology score (SAPS II) (44.0±23.1) scores, with no significant difference between groups.

**Table 2:** Relationship between comorbidities and type of patients (IM vs. ON) (N=313) IM: Internal medicine; ON: oncology. Results are expressed as number (Column percentage). ^a^P-value has been calculated using Pearson chi-square t-test. ^b^P-value has been calculated using Fisher exact test. ^c^P-value has been calculated using Fisher-Freeman-Halton exact test.

Factors	Results	Type of Patients			P-value^a^
		IM (n =148)	ON (n = 165)	Test value	
Comorbidities					
Ischemic heart disease	39 (12.5%)	27 (18.2%)	12 (7.3%)	8.609	0.003
Cerebrovascular disease	29 (9.3%)	23 (15.5%)	6 (3.6%)	13.15	<0.001
Neuromuscular disorder	14 (4.5%)	14 (9.5%)	0	-	<0.001^b^
Chronic kidney disease (CKD)	46 (14.7%)	37 (25.0%)	9 (5.5%)	23.77	<0.001
Liver cirrhosis	17 (5.4%)	13 (8.8%)	4 (2.4%)	6.143	0.013
Anemia	17 (5.4%)	13 (8.8%)	4 (2.4%)	6.143	0.013
Hypertension	133 (42.5%)	75 (50.7%)	58 (35.2%)	7.695	0.006
Immunosuppression	134 (42.8%)	16 (10.8%)	118 (71.5%)	117.43	<0.001
Performance Status (n=266)				18.019	0.002^c^
0	34 (12.8%)	22 (18.0%)	12 (8.3%)		
1	57 (21.4%)	20 (16.4%)	37 (25.7%)		
2	38 (14.3%)	11 (9.0%)	27 (18.8%)		
3	56 (21.1%)	22 (18.0%)	34 (23.6%)		
4	77 (28.9%)	44 (36.1%)	33 (22.9%)		
5	4 (1.5%)	3 (2.5%)	1 (0.7%)		

Table 3 presents initial vital signs recorded upon ICU admission. Vasopressor support was required by 238 (76%) patients, with higher usage among oncology patients (133 (80.6%) vs 105 (70.9%)), p=0.046). Mechanical ventilation was employed in 207 (66.1%) patients, also more frequently in oncology patients (118 (71.5%) vs 89 (60.1%), p=0.034). Among the 165 oncology patients, breast cancer (33, 20%) represented the most common malignancy followed by colorectal cancer (25, 15.2%) and lung cancer (12, 7.3%). Most of them had metastasis (108, 78.8%) and 122 (74%) were receiving active cancer treatment. Hematology patients accounted for 28 (17%) of the sample, the majority having lymphoma (either Hodgkin's or non-Hodgkin's) (Table 4).

**Table 3:** Relationship between clinical characteristic and type of patients (IM vs. ON) (n=313). IM: Internal medicine; ON: oncology; APACHE II, Acute physiology and chronic health evaluation; SAP II score, Simplified Acute Physiology score; GCS, Glasgow Coma Scale; MAP, mean arterial pressure; HD/CRRT, hemodialysis/continuous renal replacement therapy. Results are expressed as mean±SD, median with range, and number (percentage). ^a^P-value has been calculated using Pearson chi-square test. ^b^P-value has been calculated using independent sample t-test. ^c^P-value has been calculated using Mann Whitney U-test. ^d^P-value has been calculated using Fisher exact test.

Factors	Results	Type of Patients			P-value^a^
		IM (n=148)	ON (n=165)	Test value	
APACHE II score	25.8±16.4	24.8±18	26.8±14.9	1.056	0.292^b^
SAP II score	44.0±23.1	41.4±23.4	46.4±22.7	1.899	0.059^b^
Number of organ failure				12.161	0.054^c^
0	5 (1.6%)	4 (2.7%)	1 (0.6%)		
1	68 (21.7%)	37 (25.0%)	31 (18.8%)		
2	78 (24.9%)	38 (25.7%)	40 (24.2%)		
3	66 (21.1%)	32 (21.6%)	34 (20.6%)		
4	52 (16.6%)	17 (11.5%)	35 (21.2%)		
5	28 (8.9%)	16 (10.8%)	12 (7.3%)		
6	16 (5.1%)	4 (2.7%)	12 (7.3%)		
MAP	68.4±18.4	70.84±18.13	66.18±18.34	-2.258	0.025^b^
Heart rate	104.8±26.8	99.14±25.12	109.85±27.26	3.603	<0.001^b^
GCS	11.2 ± 4.4	11.2±4.44	11.12±4.43	-0.162	0.871^b^
ICU intervention					
Vasopressor	238 (76%)	105 (70.9%)	133 (80.6%)	3.996	0.046
Renal replacement therapy (HD/CRRT)	29 (9.3%)	23 (15.5%)	6 (3.6%)	13.151	<0.001
Invasive mechanical ventilator	207 (66.1%)	89 (60.1%)	118(71.5%)	4.511	0.034
Non-invasive mechanical ventilator	55 (17.6%)	31 (20.9%)	24 (14.5%)	2.207	0.137
Blood transfusion	69 (22%)	33 (22.3%)	36 (21.8%)	0.010	0.919
Tracheostomy	10 (3.2%)	6 (4.1%)	4 (2.4%)	-	0.526^d^

**Table 4:** Type of cancer and their treatment (N=165) Results are expressed as number (percentage). NHL: Non-Hodgkin lymphoma; HL: Hodgkin lymphoma; ALL: acute lymphocytic leukemia; AML: acute myeloid leukemia.

Factors	Results
Type of solid cancer	
Breast	33 (20%)
Colorectal	25 (15.2%)
Lung	12 (7.3%)
Oral	3 (1.8%)
Upper gastrointestinal tract	11 (6.7%)
Gynecological	12 (7.3%)
Pancreas	3 (1.8%)
Brain	2 (1.2%)
Thyroid	2 (1.2%)
Renal cell carcinoma	6 (3.6%)
Hepatobiliary	4 (2.4%)
Gall bladder	0
Ovary	6 (3.6%)
Bladder	4 (2.4%)
Other cancer	23 (13.9%)
Hematology malignancy	
NHL	6 (3.6%)
HL	6 (3.6%)
ALL	3 (1.8%)
AML	10 (6.1%)
Other	3 (1.8%)
Cancer stage (n=137)	
Non-metastasis	29 (21.2%)
Metastasis	108 (78.8%)
Site of metastasis	
Liver	43 (26.1%)
Lung	43 (26.1%)
Bone	33 (20%)
Peritoneal	20 (12.1%)
Other	36 (21.8%)
Active cancer treatment	
Chemotherapy	90 (54.5%)
Biologic	16 (9.7%)
Hormonal	10 (6.1%)
Radiation	6 (3.6%)

Outcome data

The ICU mortality rate was 151 (48.2%) and the in-hospital mortality rate was 185 (59.1)% for both groups. Despite comparable APACHE II and SAPS II scores, oncology patients had significantly higher ICU (58.8% vs. 36.5%, p<0.001) and in-hospital (72.7% vs. 43.9%, p<0.001) mortality rates with similar ICU and in-hospital length of stay. Twice as many oncology patients received Do Not Resuscitate (DNR) orders post-ICU admission (46 (27.9%) vs 26 (17.6%), p=0.03). Median ICU and hospital stays were similar, with no differences in baseline characteristics, admission reasons, or comorbidities influencing ICU mortality (Tables 5, 6).

**Table 5:** Relationship between mortality and length of stay and type of patients (IM vs. ON) (n=313). IM: Internal medicine; ON: oncology; DNR: Do Not Resuscitate. Results are expressed as mean±SD, median with range (Min.–Max.), and number (percentage). ^a^P-value has been calculated using Pearson chi-square t-test. ^b^P-value has been calculated using Mann-Whitney U-test.

Factors	Results	Type of Patients			P-value^a^
		IM (n =148)	ON (n = 165)	Test value	
ICU mortality	151 (48.2%)	54 (36.5%)	97 (58.8%)	15.54	< .001
In-hospital mortality	185 (59.1%)	65 (43.9%)	120 (72.7%)	26.78	< .001
DNR	72 (23%)	26 (17.6%)	46 (27.6%)	4.683	0.030
Length of hospital stay	12 (1-161)	12 (1-120)	12 (1-161)	12289	0.921^b^
Length of ICU stay	4 (1-156)	4 (1-92)	3 (1-156)	10935	0.108^b^

**Table 6:** Relationship between demographic, clinical characteristic and ICU mortality (n=313). Results are expressed as mean±SD, and number (percentage). CI: Confidence Interval.

Factors	Results	ICU mortality		Odds ratio (OR)	95% CI for OR	P-value
		Yes (n=151)	No (n=162)			
Comorbidities						
Ischemic heart disease						
Yes	39 (12.5%)	24 (61.5%)	15 (38.5%)	1.852	0.93-3.68	0.079
No	274 (87.5%)	127 (46.4%)	147 (53.6%)	1		
Cerebrovascular disease						
Yes	29 (9.3%)	13 (44.8%)	16 (55.2%)	0.860	0.39-1.85	0.699
No	284 (90.7%)	138 (48.6%)	146 (51.4%)	1		
Chronic kidney disease (CKD)						
Yes	46 (14.7%)	21 (45.7%)	25 (54.3%)	0.885	0.47-1.65	0.704
No	267 (85.3%)	130 (48.7%)	137 (51.3%)	1		
Liver cirrhosis						
Yes	17 (5.4%)	6 (35.3%)	11 (64.7%)	0.568	0.21-1.57	0.277
No	296 (94.6)	145 (49.0%)	151 (51.0%)	1		
Immunosuppression						
Yes	134 (42.8%)	73 (54.5%)	61 (45.5%)	1.550	0.98-2.43	0.057
No	179 (57.2%)	78 (43.6%)	101 (56.4%)	1		

None of the clinical characteristics including age, gender, blood pressure, source of ICU admission, reason for ICU admission, nor any of the comorbidities listed in Tables 6, 7 in the whole cohort correlated with ICU mortality.

**Table 7:** Relationship between demographic, clinical characteristic and ICU mortality (n=313). Results are expressed as mean±SD, and number (percentage). CI: Confidence interval.

Factors	Overall Results	ICU mortality		Odds ratio (OR)	95% CI for OR	P-value
		Yes (n =151)	No (n = 162)			
Age in years	55.93 ± 18.33	56.20±17.67	55.68±18.96	1.002	0.98-1.01	0.802
Gender						
Female	153 (48.9%)	71 (46.4%)	82 (53.6 %)	1		
Male	160 (51.1%)	80 (50.0%)	80 (50.0%)	0.866	0.55-1.35	0.525
Systolic blood pressure	103.7±27.4	102.27±29.48	105.09±25.31	0.996	0.98-1.00	0.363
Diastolic blood pressure	59.4±17.2	58.24±18.08	60.43±16.37	0.993	0.98-1.01	0.262
Source of admission						
Non-Emergency Department	92 (29.4%)	44 (47.8%)	48 (52.2%)	1		
Emergency Department	221 (70.6%)	107 (48.4%)	114 (51.6%)	1.024	0.62-1.66	0.924
Reason for ICU admission						
Respiratory failure				1.028	0.65-1.61	0.902
Yes	173 (55.3%)	84 (48.6%)	89 (51.4%)			
No	140 (44.7%)	67 (47.9%)	73 (52.1%)			
Sepsis/septic shock				1.149	0.69-1.91	0.592
Yes	232 (74.1%)	114 (49.1%)	118 (50.9%)			
No	81 (25.9%)	37 (45.7 %(	44 (54.3%)			
Neurological disorder				1.326	0.82-2.15	0.251
Yes	96 (30.7%)	51 (53.1%)	45 (46.9%)			
No	217 (69.3%)	100 (46.1%)	117 (53.9%)			
Renal dysfunction				1.176	0.70-1.97	0.538
Yes	76 (24.3%)	39 (51.3%)	37 (48.7%)			
No	237 (75.7%)	112 (47.3%)	125 (52.7%)			
Hepatic dysfunction				1.944	0.98-3.85	0.056
Yes	40 (12.8%)	25 (62.5%)	15 (37.5%)			
No	273 (87.2%)	126 (46.2%)	147 (53.8%)			

Higher APACHE II and SAP scores, vasopressor use, and mechanical ventilation rates correlated with non-survival. Venous lactate levels were elevated in oncology patients and non-survivors (Table 8). DNR status was more common among non-survivors, with a short median ICU length of stay (three days for non-survival, four days for those who survive) (Table 9).

**Table 8:** Relationship between clinical characteristic and ICU mortality (n=313). APACHE II, Acute physiology and chronic health evaluation; SAP score, Simplified Acute physiology Score; GCS: Glasgow Coma Score; MAP, mean arterial pressure; HD/CRRT, hemodialysis/continuous renal replacement therapy. Results are expressed as mean±SD, median with range, and number (percentage). CI: Confidence interval.

Factors	Results		ICU mortality		Odds ratio (OR)	95% CI for OR	P-value
			Yes (n = 151)	No (n = 162)			
APACHE II score	25.8±16.4		28.34±18.2	23.52±14.1	1.019	1.00-1.03	0.012
SAP score	44.0±23.1		47.39±24.35	40.81±21.73	1.013	1.01-1.02	0.013
Number of organ failure							0.098
0	5 (1.6%)		0 (0.0%)	5 (100.0%)	-	-	-
1	68 (21.7%)		29 (42.6%)	39 (57.4%)	0.172	0.04–0.66	0.010
2	78 (24.9%)		36 (46.2%)	42 (53.8%)	0.198	0.05-0.75	0.017
3	66 (21.1%)		28 (42.4%)	38 (57.6%)	0.170	0.04-0.65	0.010
4	52 (16.6%)		27 (51.9%)	25 (48.1%)	0.249	0.06-0.98	0.047
5	28 (8.9%)		18 (64.3%)	10 (35.7%)	0.415	0.09-1.81	0.243
6	16 (5.1%)		13 (81.3%)	3 (18.8%)	1		
MAP	68.4±18.4		66.98±19.82	69.70±16.84	0.992	0.98-1.00	0.191
GCS	11.2±4.4		11.08±4.38	11.23±4.48	0.992	0.94-1.04	0.757
ICU intervention							
Vasopressor							
Yes	238 (76.0%)		123 (51.7%)	115 (48.3%)	1.795	1.05-3.05	0.031
No	75 (24.0%)		28 (37.3%)	47 (62.7%)	1		
Renal replacement therapy (HD/CRRT)							
Yes	29 (9.3%)		14 (48.3%)	15 (51.7%)	1.001	0.46-2.15	0.997
No	284 (90.7%)		137 (48.2%)	147 (51.8%)	1		
Invasive mechanical ventilator							
Yes	207 (66.1%)		114 (55.1%)	93 (44.9%)	2.286	1.40-3.71	<0.001
No	106 (33.9%)		37 (34.9%)	69 (65.1%)	1		
Non-Invasive mechanical ventilator							
Yes	55 (17.6%)		21 (38.2%)	34 (61.8%)	0.608	0.34-1.10	0.102
No	258 (82.4%)		130 (50.4%)	128 (49.6%)	1		
Lactate (μmol/L)	2.2 (0-38.7)	2.6 (0.40-24.0)		1.9 (0.0-38.7)	1.076	1.01-1.14	0.021
Neutrophil count (10^3^/μL)	7.9 (0-88)		7.5 (0.0-50.0)	8.0 (0.0-88.0)	0.975	0.95-0.99	0.022
Serum creatinine (mmol/L)	112 (22-1828)		118 (22-950)	101.5 (33-1828)	1.000	0.99-1.001	0.539

**Table 9:** Relationship between laboratory investigation and length of stay and ICU mortality (n=313). DNR: Do Not Resuscitate. Results are expressed as mean±SD, median with range, and number (percentage). CI: Confidence interval.

Factors	Results	ICU mortality		Odds ratio (OR)	95% CI for OR	P-value
		Yes (n=151)	No (n=162)			
Microbiology						
Bacterial infection						
Yes	91 (29.1%)	43 (47.3%)	48 (52.7%)	0.946	0.58-1.54	0.822
No	222 (70.9%)	108 (48.6%)	114 (51.4%)	1		
Fungal infection						
Yes	17 (5.4%)	11 (64.7%)	6 (35.3%)	2.043	0.73-5.67	0.170
No	296 (94.6%)	140 (47.3%)	156 (52.7%)	1		
Blood stream infection						
Yes	116 (37.1%)	60 (51.7%)	56 (48.3%)	1.248	0.78-1.97	0.345
No	197 (62.9%)	91 (46.2%)	106 (53.8%)	1		
Lung infection						
Yes	83 (26.5%)	46 (55.4%)	37 (44.6%)	1.480	0.89-2.45	0.128
No	230 (73.5%)	105 (45.7%)	125 (54.3%)	1		
Urine infection						
Yes	35 (11.2%)	19 (54.3%)	16 (45.7%)	1.313	0.64-2.65	0.448
No	278 (88.8%)	132 (47.5%)	146 (52.5%)	1		
DNR						
Yes	72 (23.0%)	43 (59.7%)	29 (40.3%)	1.826	1.07-3.12	0.027
No	241 (77.0%)	108 (44.8%)	133 (55.2%)	1		
Length of hospital stay	72 (23%)	11.0 (1 –161)	13(1 – 101)	0.999	0.98-1.01	0.817
Length of ICU stay	1 (1 – 161)	3(1–156)	4(1–92)	1.010	0.99-1.03	0.282

Multiple logistic regression analysis identified two significant predictors of ICU mortality: cancer diagnosis and ischemic heart disease (Table 10). Invasive mechanical ventilation specifically predicted ICU mortality in cancer patients (P=0.002, adjusted odds ratio (AOR): 3.76; CI: 1.61-8.77) (Table 11), while DNR status predicted ICU mortality in non-cancer patients (P=0.005, AOR: 3.97; CI: 1.53-10.29) (Table 12).

**Table 10:** Multiple logistic regression analysis to identify factor associated with ICU mortality after adjusting for confounders among oncology patients (n=165). DNR: Do Not Resuscitate; AOR: adjusted odds ratio, CI: confidence interval. Multiple logistic regression analysis to identify factors associated with ICU mortality after adjusting for confounders. Hosmer and Lemshow chi-square=11.336, p= 0.183 (indicating adequate fit of the model to the data).

Factors	ICU Mortality		
	AOR	95% CI for AOR	P-value
Age in years	1.013	0.990-1.037	0.260
Source of admission			
Non-Emergency Department	1		
Emergency Department	1.496	0.702-3.186	0.297
Reason for ICU admission			
Sepsis/septic shock	0.750	0.297-1.895	0.543
Comorbidities			
Ischemic heart disease	3.164	0.57-17.582	0.188
APACHE II score	1.003	0.977-1.029	0.831
MAP score	0.990	0.969-1.012	0.371
Heart rate	1.014	0.999-1.029	0.066
ICU intervention			
Vasopressor	0.801	0.300-2.141	0.658
Renal replacement therapy (HD/CRRT)	0.394	0.059-2.623	0.336
Invasive mechanical ventilator	3.761	1.613-8.771	0.002
Serum albumin	1.050	0.991-1.111	0.098
DNR	0.801	0.360-1.785	0.587

**Table 11:** Multiple logistic regression analysis to assess the relationship between Type (IM vs. Oncology) with ICU mortality after adjusting for confounders (n=313). DNR: Do Not Resuscitate; AOR: adjusted odds ratio, CI: confidence interval. Multiple logistic regression analysis to assess the relationship between Type (IM vs. Oncology) with ICU mortality after adjusting for confounders (age, source of admission, reason for ICU admission - sepsis, septic shock, ischemic heart disease, APACHE II score, MAP score, heart rate, vasopressor, renal replacement therapy (HD/CRRT), invasive mechanical ventilator, serum albumin, and DNR). Hosmer and Lemshow chi-square=7.893, P=0.444 (indicating adequate fit of the model to the data). IM: Internal medicine; ON: oncology; APACHE II, Acute physiology and chronic health evaluation; SAP II score, Simplified Acute Physiology score; MAP, mean arterial pressure; HD/CRRT, hemodialysis/continuous renal replacement therapy.

Factors	ICU Mortality		
	AOR	95% CI for AOR	P-value
Type			
IM	1		
Oncology	2.504	1.481-4.233	<0.001
Age in years	1.004	0.990-1.018	0.565
Source of admission			
Non-Emergency Department	1		
Emergency Department	1.105	0.644-1.896	0.718
Reason for ICU admission			
Sepsis/septic shock	0.877	0.486-1.583	0.664
Comorbidities			
Ischemic heart disease	2.187	1.018-4.699	0.045
APACHE II score	1.012	0.996-1.028	0.150
MAP score	1.000	0.986-1.015	0.993
Heart rate	1.004	0.995-1.014	0.404
ICU intervention			
Vasopressor	1.229	0.630-2.398	0.545
Renal replacement therapy (HD/CRRT)	1.021	0.434-2.404	0.962
Invasive mechanical ventilator	1.710	0.968-3.021	0.065
Serum albumin	1.002	0.964-1.041	0.929
DNR	1.574	0.884-2.801	0.123

**Table 12:** Multiple logistic regression analysis to identify factor associated with ICU mortality after adjusting for confounders among IM patients (n=148). DNR: Do Not Resuscitate; AOR: adjusted odds ratio, CI: confidence interval; APACHE II, Acute physiology and chronic health evaluation; SAP II score, Simplified Acute Physiology score; MAP, mean arterial pressure; HD/CRRT, hemodialysis/continuous renal replacement therapy. Multiple logistic regression analysis to identify factors associated with ICU mortality after adjusting for confounders. Hosmer and Lemshow Chi-Square=7.45, p= 0.488 (indicating adequate fit of the model to the data).

Factors	ICU Mortality		
	AOR	95% CI for AOR	P-value
Age in years	1.003	0.985-1.022	0.761
Source of admission			
Non-Emergency Department	1		
Emergency Department	0.981	0.388-2.175	0.846
Reason for ICU admission			
Sepsis/septic shock	0.969	0.407-2.305	0.943
Comorbidities			
Ischemic heart disease	1.830	0.728-4.601	0.199
APACHE II score	1.010	0.988-1.033	0.356
MAP score	1.001	0.979-1.024	0.921
Heart rate	0.998	0.983-1.013	0.775
ICU intervention			
Vasopressor	1.749	0.611-5.006	0.297
Renal replacement therapy (HD/CRRT)	1.522	0.563-4.114	0.408
Invasive mechanical ventilator	0.850	0.357-2.024	0.713
Serum albumin	0.969	0.914-1.027	0.290
DNR	3.972	1.53-10.282	0.005

## Discussion

This retrospective cohort study enrolled critically ill patients admitted for medical reasons, characterized by high acuity evidenced by high APACHE II and SAP scores. We compared outcomes between patients with solid (the majority had metastasis)/hematological malignancies and non-cancer patients, providing valuable insights into two patient groups known for their prolonged ICU stay and poor prognoses due to complex diseases and associated comorbidities. Notably, this study's control group and focused approach differentiate it from prior local studies [17,21].

The study found significantly distinct differences between cancer and non-cancer ICU patients. Cancer patients were younger, had lower blood pressure upon admission, and were more prone to sepsis and septic shock due to their immunocompromised status. They required more intensive interventions like vasopressors and mechanical ventilation, but less renal replacement therapy, which is likely due to the patient’s hemodynamic instability and clinician judgment regarding the futility of renal replacement therapy. Cancer patients faced higher ICU and in-hospital mortality rates and were more likely to receive Do-Not-Resuscitate orders.

ICU mortality rates for cancer patients vary globally and across studies. Our findings mirror those of Olaechea Astigarraga et al., which reported higher mortality among cancer patients compared to non-cancer patients. However, the overall mortality rate in their study was significantly lower than ours (12.3% vs 58.8%) [22]. Similarly, Yuan et al. observed comparable ICU lengths of stay between cancer and non-cancer patients, but significantly higher mortality rates among cancer patients up to three years post-ICU admission. Also, their overall mortality rate was substantially lower than ours (28-day mortality, 18.7%; three-year mortality, 51.7%) [25]. Rugaan et al. [17] reported ICU and hospital mortality rates of 32.4% and 47.3%, respectively, for cancer patients admitted to the ICU, while AlSaied et al. [21] reported ICU and one-year mortality rates of 52% and 85%, respectively, while 48% of them had a hematological malignancy. Notably, our cohort had higher APACHE II scores compared to Rugaan et al. and Alsaied et al. (25.8 vs 19.8 and 21.9, respectively) and required more vasopressors (80.6% vs 50% and 67%, respectively) and mechanical ventilation (71.5% vs 50.8% and 73% respectively), indicating greater illness severity; also 78.8% of the oncology group presented with metastasis compared to 43% in the study by Rugaan et al. Despite higher mortality rates in the cancer group, ICU and hospital lengths of stay remained unchanged.

Preliminary analysis identified several factors associated with higher ICU mortality in both cancer and non-cancer patients, including higher APACHE II (P=0.012) and SAP scores (P=0.013). ICU interventions like vasopressor support (P=0.031) and invasive mechanical ventilation (P<0.001), elevated liver enzymes, and Do-Not-Resuscitate (DNR) orders (Table 8, 9). However, multiple logistic regression analysis revealed only two significant predictors of mortality: cancer diagnosis and ischemic heart disease. Notably, DNR decisions, age, admission source, vital signs, ICU admission reasons, and interventions did not predict mortality (Table 10). Interestingly, ischemic heart disease was more common among non-cancer patients, potentially understating the mortality difference. These results are consistent with the findings of Rugaan et al. and AlSaied et al., which demonstrated that organ dysfunction scores (APACHE II, SAP, SOFA) and the need for organ support therapies (invasive mechanical ventilation, vasopressors, and renal replacement therapy) were significant predictors of mortality. A Jordanian study reported a 48.9% ICU mortality rate among cancer patients with septic shock, identifying mechanical ventilation, APACHE II, lactic acidosis, high bilirubin, thrombocytopenia, and positive cultures as significant mortality predictors [26]. Van der Zee et al. reported a higher ICU and one-year mortality in the cancer group compared to the non-cancer group. Again, the reported ICU mortality was much less compared to our results (28.6% for hematological, 13.6% for solid cancer, and 12.5% for non-cancer; P<0.01)[24].

Our study contradicts Le Borgne et al.'s French trial, which found no mortality or length-of-stay differences between septic shock patients with and without malignancy [27]. Despite our study's higher proportion of septic shock cases in the cancer group, multiple logistic regression analysis confirmed significantly higher mortality rates among cancer patients. By shedding light on the outcomes of critically ill patients with cancer, this study enhances our understanding of this complex patient group. The findings offer valuable guidance for oncologists and intensivists, supporting informed decision-making and resource prioritization. Furthermore, this research provides a foundation for future studies, quality improvement initiatives, and educational programs aimed at optimizing care for critically ill patients with cancer.

Study strengths and limitations

The study's strengths include sample size calculation, focused cohort selection, control group and regression analysis. However, limitations include: its retrospective observational design, small sample size, single-center setup with narrow admission criteria, and control group limitations. Additionally, results may not generalize to early-stage cancer patients, less acute cases, or diverse populations, as our cohort primarily focused on patients with advanced-stage cancer.

## Conclusions

Critically ill cancer patients admitted to ICU face higher mortality rates compared to non-cancer patients despite similar lengths of stay. Invasive mechanical ventilation emerges as a key predictor of mortality among cancer patients. Conversely, cancer diagnosis predicts mortality across both groups. These findings underscore the importance of discussing end-of-life care goals with advanced-stage cancer patients requiring mechanical ventilation.
